# Cloflucarban Illuminates Specificity and Context-Dependent Activation of the PINK1–Parkin Pathway by Mitochondrial Complex Inhibition

**DOI:** 10.3390/biom14030248

**Published:** 2024-02-20

**Authors:** Adrian T. Ramirez, Zeyu Liu, Quanbin Xu, Sarah Nowosadtko, Xuedong Liu

**Affiliations:** Department of Biochemistry, Jennie Smoly Caruthers Biotechnology Building, University of Colorado-Boulder, 3415 Colorado Ave, Boulder, CO 80303, USA; adrian.ramirez@colorado.edu (A.T.R.); quanbin.xu@colorado.edu (Q.X.); sarah.nowosadtko@colorado.edu (S.N.)

**Keywords:** CCCP, cloflucarban, electron transport complex III, complex V, bovine serum albumin, human serum albumin, mitophagy, mitochondrial quality control, Parkin, PINK1

## Abstract

The PTEN-induced kinase 1 (PINK1)-Parkin pathway plays a vital role in maintaining a healthy pool of mitochondria in higher eukaryotic cells. While the downstream components of this pathway are well understood, the upstream triggers remain less explored. In this study, we conducted an extensive analysis of inhibitors targeting various mitochondrial electron transport chain (ETC) complexes to investigate their potential as activators of the PINK1–Parkin pathway. We identified cloflucarban, an antibacterial compound, as a novel pathway activator that simultaneously inhibits mitochondrial complexes III and V, and V. RNA interference (RNAi) confirmed that the dual inhibition of these complexes activates the PINK1–Parkin pathway. Intriguingly, we discovered that albumin, specifically bovine serum albumin (BSA) and human serum albumin (HSA) commonly present in culture media, can hinder carbonyl cyanide m-chlorophenyl hydrazone (CCCP)-induced pathway activation. However, cloflucarban’s efficacy remains unaffected by albumin, highlighting its reliability for studying the PINK1–Parkin pathway. This study provides insights into the activation of the upstream PINK1–Parkin pathway and underscores the influence of culture conditions on research outcomes. Cloflucarban emerges as a promising tool for investigating mitochondrial quality control and neurodegenerative diseases.

## 1. Introduction

The PTEN-induced kinase 1 (PINK1)-Parkin signaling pathway helps in maintaining mitochondrial quality control and is implicated in neurodegenerative diseases like Parkinson’s disease [1,2,3]. Under normal conditions, PINK1 is imported into mitochondria but cleaved by PINK1/PGAM5-associated rhomboid-like protease (PARL) located in the inner mitochondrial membrane [4,5,6]. Cleaved PINK1 is released into the cytosol and rapidly degraded via the proteasome [7]. When mitochondria are damaged and consequently lose mitochondrial membrane potential (ΔΨm), PINK1 import is halted. This results in the accumulation of full length PINK1 and autoactivation via autophosphorylation on the outer mitochondrial membrane (OMM) [8,9]. PINK1 then phosphorylates Parkin, an E3 ubiquitin ligase, to recruit it to mitochondria to initiate mitophagy [10,11,12,13]. Activated PINK1 also phosphorylate ubiquitin at serine 65, which further activates Parkin and serves as a signal for recruiting other mitophagy-related proteins [14,15,16,17]. Moreover, PINK1 can phosphorylate MFN2, a key regulator of mitochondrial fusion, leading to its ubiquitination and degradation to limit damaged mitochondria fusing [18,19,20]. Upon activation, Parkin ubiquitylates many proteins on the OMM [21,22,23], resulting in the recruitment of autophagy receptors including optineurin (OPTN), NDP52/(coiled–coiled domain-containing protein), and the Tax-1-binding protein (TAX1BP1) [24,25]. TANK-binding kinase 1 (TBK1) phosphorylates OPTN, leading to its enhanced binding to microtubule-associated protein 1A/1B-light chain 3A (LC3 A) and the formation of autophagosome, leading to the elimination of damaged mitochondria through mitophagy, a mitochondria-specific form of autophagy [25,26]. Numerous studies have underscored the importance of the PINK1–Parkin pathway as mutations in PINK1 and Parkin genes are shown to cause early-onset familial Parkinson’s disease (PD) [27,28]. Given the critical role of the PINK1–Parkin pathway in preventing PD, a comprehensive understanding of this pathway is essential [29].

While the downstream components of the PINK1–Parkin pathway have been well characterized, the upstream signals that trigger this pathway activation remain less elucidated. Youle and coworkers first reported that the protonophore carbonyl cyanide m-chlorophenyl hydrazone (CCCP) can trigger Parkin translocation to the mitochondria outer membrane, which promotes mitophagy [30]. CCCP is a lipophilic protonophore capable of transporting protons across biological membranes, thereby collapsing the proton gradient. Disrupting the mitochondrial membrane potential (ΔΨm) by dissipating the proton gradient across the IMM, CCCP uncouples oxidative phosphorylation and halts ATP synthesis [31]. CCCP has been widely used as a tool to study the underlining mechanisms of the PINK1–Parkin pathway activation and mitophagy [31]. However, the ability of CCCP to trigger PINK1 and the subsequent Parkin mitochondrial recruitment varies with cell types or culture conditions, making it difficult to generalize observations made in one system to another [32]. Although it is widely accepted that mitochondrial damage or depolarization will activate this pathway, many mitochondrial toxins are capable of depolarizing Δψm but fail to trigger Parkin mitochondrial recruitment [33]. This raises the question of which specific types of mitochondrial toxins are effective in triggering the PINK1–Parkin pathway. Additionally, the endogenous signals that activate this pathway have not been fully enumerated. It has been shown that the expression of misfolded proteins or the inhibition of chaperons in the mitochondrial matrix can stimulate Parkin mitochondrial localization without depolarizing Δψm [34,35,36,37]. Damages to mtDNA may also serve as an endogenous signal. Increases in Parkin translocation to mitochondria were observed in cells overexpressing a mutated Twinkle, a mitochondrial helicase known to disrupt mtDNA replication and produce mtDNA deletions [38]. Furthermore, Parkin or PINK1 knockout mice accumulate mtDNA mutations following exhaustive exercise and activate stimulator of interferon genes (STING)-dependent innate immune responses [39]. Despite these discoveries, many unanswered questions remain regarding other endogenous signals that are sufficient to activate Parkin.

In the present study, we profiled a variety of inhibitors known to target different mitochondrial electron transport chain (ETC) complexes. We found that compounds targeting both complex III and complex V serve as potent inducers of Parkin recruitment to mitochondria. Specifically, we have identified cloflucarban, an antibacterial small molecule, as a novel activator of the PINK1–Parkin pathway due to its inhibition of both mitochondrial complex II/III and complex V [40]. Using short-hairpin RNA (shRNA) to knock down each of these complexes individually and in combination with a single complex inhibition, we confirmed that simultaneous inhibition activates the PINK1–Parkin pathway endogenously. This suggests that deficiencies in complexes III and V may act as triggers for pathway activation. We also unveiled that the presence of bovine serum albumin (BSA) or human serum albumin (HSA) in the culture medium inhibits the CCCP-induced activation of the PINK1–Parkin pathway, whereas cloflucarban remains unaffected by albumin. Building on our previous findings that glucose concentrations in culture media affect CCCP-induced PINK1–Parkin activation [41], this study highlights another key cell culture component, BSA/HSA, that influences the consistency of research using CCCP. Following the suggestions from other works, our findings confirm that the PINK1–Parkin pathway specifically responds to perturbations involving both complex III and V when they are combined [42,43]. Cloflucarban emerges as a potent and reliable activator for studying the PINK1–Parkin pathway, especially when compared to CCCP. Interestingly, CCCP’s effectiveness can be compromised by the presence of HSA/BSA in the culture medium, while cloflucarban remains consistent.

## 2. Results

### 2.1. Profiling the Activity of Electron Transport Chain Inhibitors on Activation of the PINK1–Parkin Pathway

Numerous studies indicate that the PINK1–Parkin pathway plays a key role in the selective degradation of damaged or dysfunctional mitochondria. Based on this, one would expect that inhibitors of ETC, known for causing mitochondrial damage, to act as potent activators of this pathway, similar to CCCP. To explore and validate this hypothesis, we conducted an ETC inhibitor screen using various drugs known to impair mitochondrial function. These drugs included simvastatin, nicardipine, troglitazone, ciglitazone, SB-224289, trans-resveratrol, myxothiazole, and cloflucarban [44,45,46,47,48,49]. We assessed the impact of these compounds on mitochondrial ETC complexes using the MitoTox Complete OXPHOS assay kit. This kit offers five separate assays utilizing isolated mitochondrial complexes in vitro to gauge how each compound influences individual complex activities. Alongside the small molecules mentioned above, rotenone, thenoyltrifluoroacetone (TTFA), antimycin A, potassium cyanide (KCN), and oligomycin A were employed as positive controls for each complex as they are known inhibitors [50,51,52,53,54].

**Figure 1:**
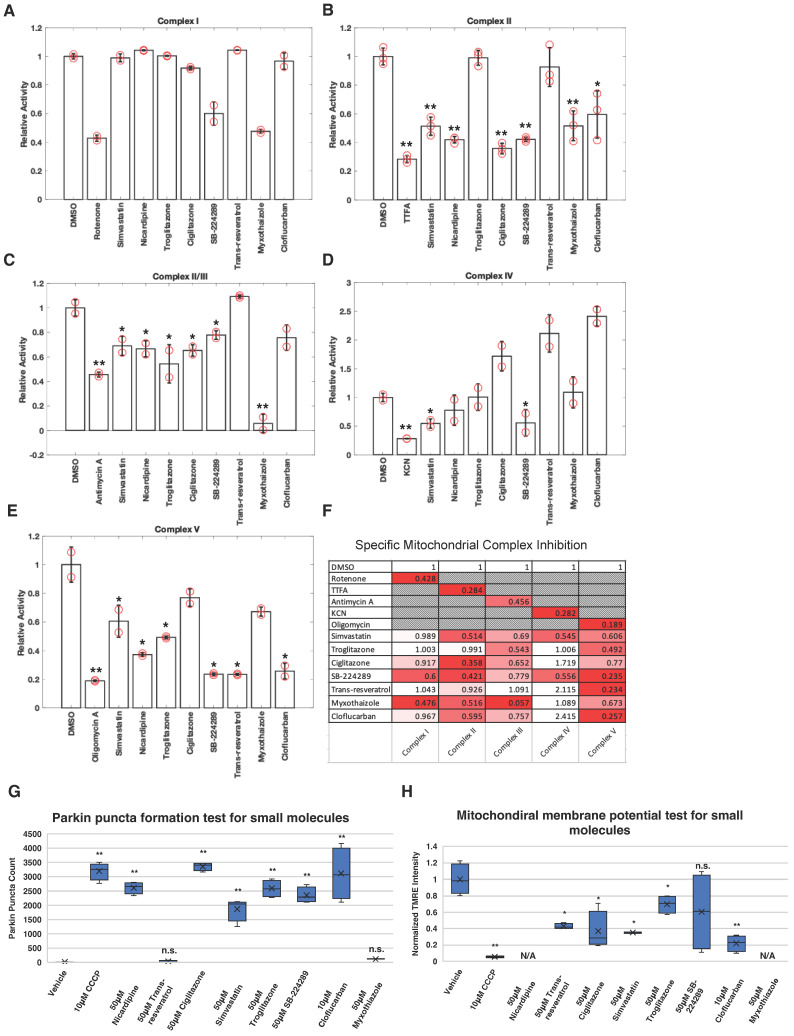
Profile of inhibition of mitochondrial complex activity by small molecules and their effects on Parkin mitochondrial recruitment. (**A**–**E**) Activity of mitochondrial complexes I–V were measured using in vitro mitochondrial complex activity assay kits following the instructions of the manufacturers. Kits for individual complexes were the treated vehicle, 10 µM of specific complex inhibitor indicated on graph, or 50 µM of indicated compound of interest following the instruction of the manufacturer (Abcam). Change in activity was normalized to vehicle control-treated samples. Two or three independent measurements were taken as indicated on the graphs. Error bars represent mean ± SE. (**F**) Average changes in activity across the five complexes were tabulated for each compound to illustrate the profile of inhibition of mitochondrial complex activities by the small molecules. The data are presented as the average of duplicate or triplicate measurements and is normalized as a percentage relative to the DMSO control. A color code has been assigned, ranging from white to red gradient, with normalized activity after drug addiction, represented as white and the lowest value for each complex displayed as red. (**G**) For each condition, four wells of 5000 HeLa Venus-Parkin RFP-MTS-Smac cells were treated with vehicle, CCCP, or indicated compound and imaged for 14 h using a high content confocal microscope Phenix Opera. Total number of Parkin puncta was quantified using method#2 mentioned in method section for every 30 min. For each drug, the time point with the highest average puncta number was selected for presentation purpose (*n* = 4). (**H**) For each condition, four wells of 5000 HeLa C9 cells were incubated with TMRE dye, and then cells were treated with vehicle, CCCP, or indicated compounds for 1 h. TMRE fluorescence was imaged before and after 1 h drug incubation and quantified for the change in fluorescent intensity over that time period. Data were further normalized using intensity at 1 h divided by intensity at 0 h (*n* = 4). Error bars represent mean ± SD. Student’s *t*-test was used for statistical analysis of data. N/A = not applicable. * *p* < 0.05, ** *p* < 0.01 and n.s. > 0.05.

As expected, SB-224289 and myxothiazole reduced complex I activity to 60% and 48% relative to the vehicle, respectively (Figure 1A). Simvastatin, ciglitazone, SB-224289, myxothiazole, and cloflucarban inhibited complex II activity by 51%, 36%, 42%, 52%, and 60%, respectively (Figure 1B). Similarly, reductions in complex III activity were observed: simvastatin, troglitazone, ciglitazone, SB-224289, myxothiazole, and cloflucarban decreased it by 69%, 54%, 65%, 78%, 6%, and 76%, respectively (Figure 1C). Complex IV activity was inhibited by simvastatin and SB-224289 by 55% and 56%, respectively (Figure 1D). Lastly, complex V activity was reduced by simvastatin, troglitazone, ciglitazone, SB-224289, trans-resveratrol, myxothiazole, and cloflucarban by 60%, 49%, 77%, 24%, 23%, 67%, and 26%, respectively (Figure 1E). These data offer a comprehensive profile detailing how these small molecules inhibit mitochondrial complex activity (Figure 1F).

Subsequently, we evaluated the impact of each drug on the PINK1–Parkin pathway by monitoring the recruitment of Parkin to mitochondria following drug treatment. HeLa cells expressing RFP-MTS-Smac and Venus-Parkin-WT were exposed to 50 μM of each indicated compound, with dimethyl sulfoxide (DMSO) as a vehicle control and 10 μM CCCP as a positive control. Parkin puncta counts were scored via live cell imaging over a 14 h period, 30 min per capture between 0 to 2 h and 4 h per capture between 2 to 14 h. For each compound, we selected the time capture period with the highest number of Parkin puncta to facilitate interpretation, accounting for varying compound dynamics (refer to Figure 1G and Supplemental Figure S1H). Remarkably, all compounds, except for trans-resveratrol and myxothiazole, induced some degree of the recruitment of Parkin to mitochondria. This observation suggests a potential correlation between ETC inhibition and the activation of the PINK1–Parkin pathway.

To further investigate whether the mitochondrial recruitment of Parkin induced by each compound resulted from the dissipation of Δψm, we monitored Δψm using tetramethylrhodamine ethyl ester (TMRE) staining, a dye that accumulates in healthy, energized mitochondria [55]. HeLa C9 cells were pre-treated with 2 µM of TMRE for 30 min, washed with Dulbecco’s phosphate-buffered saline (DPBS), and then exposed to DMSO, CCCP, or the indicated compounds for 1 h. TMRE fluorescence after 1 h was compared to the initial levels. As expected, CCCP decreased TMRE fluorescence, confirming that its induction of Parkin recruitment is due to Δψm dissipation (Figure 1H). A similar pattern was observed for ciglitazone, simvastatin, troglitazone, SB-224289, and cloflucarban, suggesting that they induce Parkin colocalization at least partially by dissipating Δψm. We were not able to assess the effect of nicardipine and myxothiazole on mitochondrial membrane potential because of their autofluorescence interfering TMRE readouts. Among all tested compounds, cloflucarban was the most effective at triggering Parkin’s recruitment to mitochondria. Therefore, we conducted further investigations to elucidate its mechanism of action in activating the PINK1–Parkin pathway.

### 2.2. Cloflucarban Induces Mitophagy by Recruiting LC3

**Figure 2:**
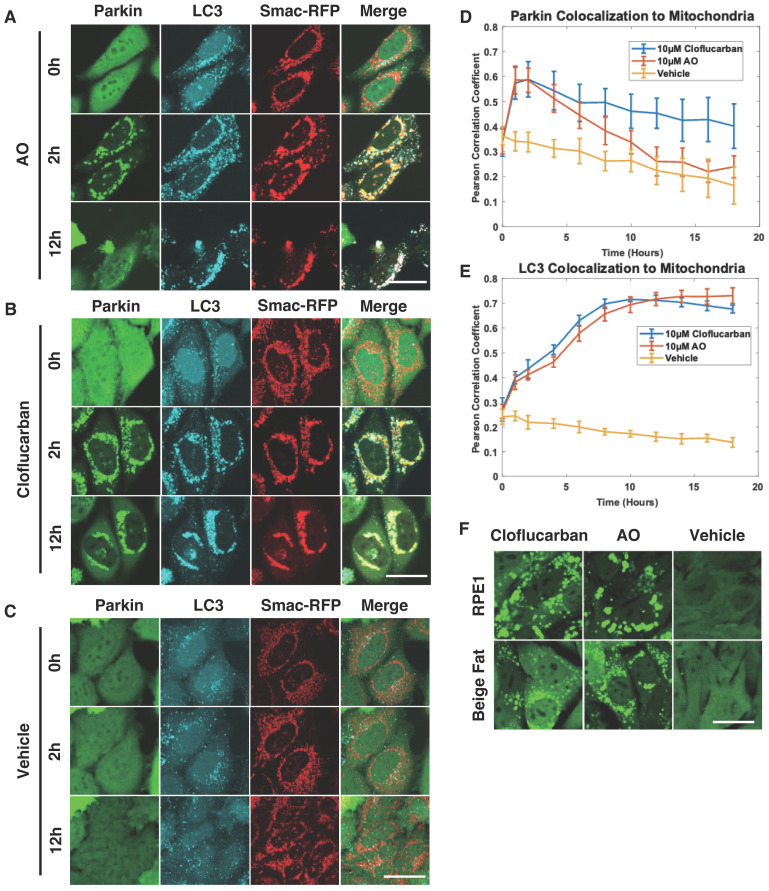
Cloflucarban causes Parkin and LC3 colocalization to mitochondria. Four wells of 5000 HeLa Venus-Parkin RFP-MTS-Smac LC3-CFP cells were treated with either (**A**) 10 µM of antimycin and oligomycin (AO), (**B**) 10 µM of cloflucarban, or (**C**) vehicle control for the indicated amount of time and imaged for YFP, RFP, and CFP signal. (**D**) Pearson’s colocalization coefficient between RFP-MTS-Smac and Venus-Parkin was measured to quantify colocalization of Parkin to mitochondria using method#1 mentioned in method section (*n* = 4). (**E**) Pearson’s colocalization coefficient between RFP-MTS-Smac and LC3-CFP was measured to quantify colocalization of Parkin to mitochondria (*n* = 4). (**F**) RPE1 and beige fat cells expressing Venus-Parkin were treated with 10 µM of cloflucarban, 10 µM of AO, or vehicle control for 2 h and imaged. Error bars represent mean ± SD. Scale bar = 50 µm.

Given that mitophagy initiated by Parkin colocalization is generally followed by LC3 recruitment [56], we further investigated whether cloflucarban would conform to this established pattern. HeLa cells expressing RFP-Smac-MTS (a mitochondrial marker), Venus-Parkin, and CFP-LC3 were treated with either 10 µM of cloflucarban or a combination of 10 µM of AO, which served as a positive control due to their known complex III and complex V inhibitory effect on Parkin recruitment. DMSO was used as a vehicle control. We employed live cell imaging to monitor the colocalization of both Parkin and LC3 with mitochondria over an 18 h period (Figure 2). Parkin’s colocalization with mitochondria was measured using Pearson’s correlation coefficient. As expected, AO treatment induced Parkin translocation to mitochondria, followed by LC3 recruitment (Figure 2A). In cells treated with cloflucarban, we observed a similar pattern of Parkin and LC3 recruitment to mitochondria (Figure 2B), compared to the vehicle treatment (Figure 2C). One noteworthy observation was that the presence of Parkin on mitochondria diminished more rapidly following AO treatment (Figure 2D). The reduction in the mitochondrial Parkin signal after its initial recruitment is generally attributed to the subsequent mitochondrial degradation and Parkin’s consequent redistribution back to the cytosol. This suggests that mitophagy proceeds more swiftly with AO treatment. Interestingly, the kinetics and trends of LC3 colocalization with the mitochondrial signal were similar for both cloflucarban and AO treatments (Figure 2E). This suggests that AO may facilitate faster mitochondrial disassembly, leading to quicker Parkin redistribution to the cytosol compared to cloflucarban.

To explore the generalizability of these findings, we examined Parkin puncta formation in other cell types, specifically human retinal pigment epithelial cells (RPE1) and beige fat cells, which also expressed Venus-Parkin. Both cell types were treated with DMSO, AO, or cloflucarban, and Parkin puncta formation was assessed (Figure 2F). As with HeLa cells, robust Parkin puncta formation was observed in both RPE1 and beige fat cells upon treatment with either AO or cloflucarban. This suggests that the mechanism by which cloflucarban induces Parkin puncta formation is broadly applicable across different cell types.

### 2.3. Cloflucarban-Induced Parkin Mitochondrial Translocation Is PINK1-Dependent

**Figure 3:**
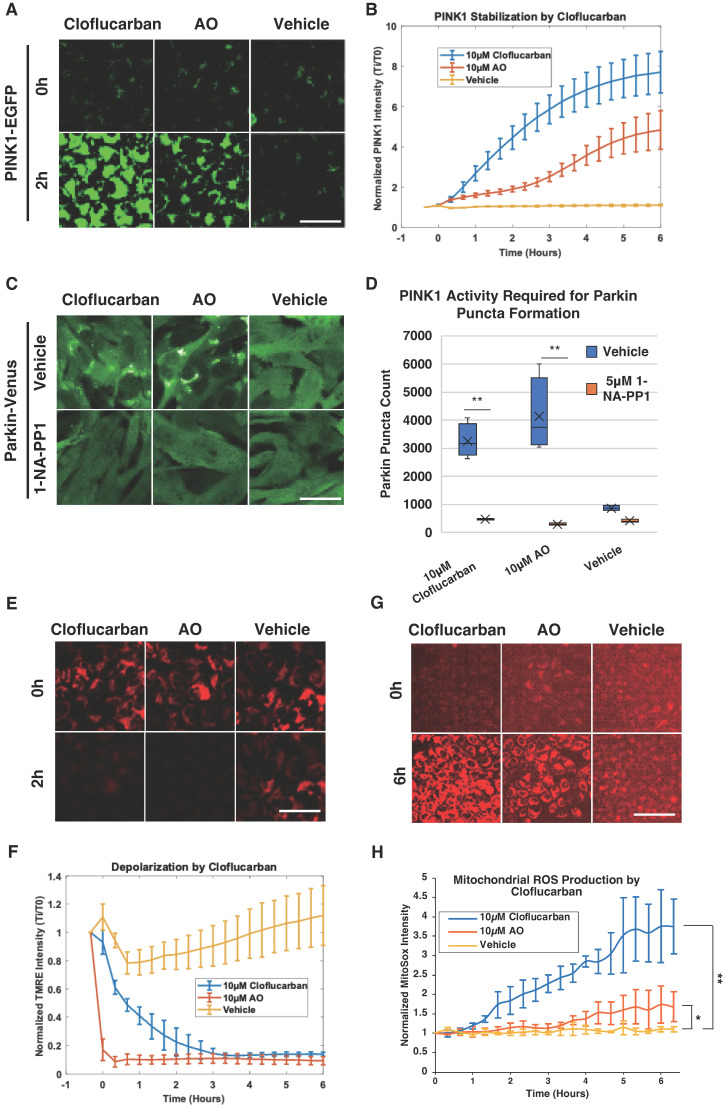
PINK1 is stabilized by cloflucarban and is required for Parkin activity. (**A**) Four wells of 5000 HeLa PINK1-EGFP cells were treated with 10 µM of cloflucarban, 10 µM of AO or vehicle control and then imaged every 20 min. Representative images of PINK1-EGFP accumulation from mitochondrial damage shown for the three conditions at 0 and 2 h post treatments. Scale bar = 100 µm. (**B**) Quantification of PINK1 accumulation quantified by measuring EGFP signal intensity and normalizing to initial EGFP determined for each condition (*n* = 4). (**C**) For each condition, four wells of 5000 MEF Parkin-Venus PINK1-/-PINK1-M318A cells were initially treated with vehicle (top row) or 5 µM of 1−NA−PP1 (bottom row) for 20 min. Cells were then treated with either vehicle, 10 µM of AO or 10 µM of cloflucarban for 2 h and then imaged for Parkin-YFP puncta formation. Scale bar = 50 µm. (**D**) Quantification of Parkin puncta formation at 2 h of drugs treatment (*n* = 4). (**E**) Four wells of 5000 HeLa C9 cells were stained with TMRE dye and then treated with 10 µM of cloflucarban, 10 µM of AO, or vehicle control. TMRE signal imaged every 10 min, and shown are representative images of TMRE signal for the indicated conditions at 2 h. Scale bar = 100 µm. (**F**) Depolarization was quantified by normalizing the TMRE signal to the signal measured before treatment for each condition (*n* = 4). (**G**) Four wells of 5000 HeLa C9 cells were stained with MitoSox red dye and then treated with 10 µM of cloflucarban, 10 µM of AO, or vehicle control. MitoSox signal imaged every 20 min, and shown are representative images of MitoSox signal for the indicated conditions at 6 h. Scale bar = 200 µm. (**H**) MitoSox signal was quantified by normalizing at initial signal for each condition. Error bars represent mean ± SD. Student’s *t*-test was used for statistical analysis of data (*n* = 4). * *p* < 0.05 and ** *p* < 0.01.

As PINK1 is known to function upstream of Parkin activation, we sought to determine whether cloflucarban-induced Parkin translocation is PINK1-dependent. HeLa cells expressing PINK1-EGFP were treated with 10 µM of cloflucarban, 10 µM of AO, or DMSO as a vehicle control. The EGFP signal was monitored for six hours using live cell imaging. HeLa cells did not express endogenous Parkin. As expected, the PINK1-EGFP signal increased steadily with AO treatment (Figure 3A), while no significant change was observed with the vehicle control. Under these experimental conditions, cloflucarban induced a more robust elevation of PINK1-EGFP than AO (Figure 3A,B). This suggests that cloflucarban most likely activates Parkin translocation by stimulating PINK1, similar to the action of AO.

To further evaluate the PINK1-dependency of cloflucarban-induced Parkin translocation, we engineered an MEF cell line expressing Venus-Parkin with a PINK1 knockout and subsequently rescued it with the PINK1-M318A mutant. This analog-sensitive mutant allows for the transient inhibition of PINK1 kinase activity through treatment with the small molecule 1-NA-PP1 [57]. These engineered cells were treated with cloflucarban, AO, or a vehicle, with or without 5 µM of 1-NA-PP1 as a pre-treatment. Both cloflucarban and AO triggered Parkin colocalization in the absence of 1-NA-PP1; however, neither treatment was effective in inducing Parkin translocation when 1-NA-PP1 was present (Figure 3C,D). It is worth noting that PINK1-M318A exhibited reduced activity compared to its wild-type counterpart, as observed with many other analog-sensitive kinases. The number of Parkin puncta in MEF cells was lower than that in HeLa cells. Nevertheless, these results confirm that cloflucarban-induced Parkin translocation is PINK1-dependent and occurs through the activation of the PINK1–Parkin pathway.

Given that Δψm dissipation is a major mechanism for PINK1 stabilization and that the loss of Δψm leads to an increase in reactive oxygen species (ROS) [17,58], we examined whether cloflucarban induces Δψm loss and ROS formation. To do this, HeLa C9 cells were pre-treated with 2 µM of TMRE for 30 min, washed with DPBS, and then treated with 10 µM of cloflucarban, 10 µM of AO, or DMSO. TMRE signals were monitored over six hours, with captures taken at 20 min intervals. Additionally, for ROS detection, HeLa C9 cells were pre-treated with 500 nM MitoSox for 30 min, washed, and then treated as before. MitoSox signals were captured over an hour, with 30 min intervals between captures. Our results indicated that both AO and cloflucarban induced Δψm depletion, although the effect was slower with cloflucarban (Figure 3E,F). Furthermore, an increase in ROS levels was observed in both AO- and cloflucarban-treated cells (Figure 3G,H). These observations lend further support to our conclusion that cloflucarban activates the PINK1–Parkin pathway by dissipating Δψm and elevating ROS, similar to the action of AO.

### 2.4. Complex III and V Knockdowns Confirm the Activation of PINK1–Parkin Pathway Occurs via Dual Inhibition

In light of the observed correlation between cloflucarban’s dual inhibition of mitochondrial complexes III and V and the activation of the PINK1–Parkin pathway, we aimed to determine whether this dual inhibition serves as the causative factor for pathway activation or if it might be an unintended off-target effect of cloflucarban. To establish this causal relationship, we employed an orthogonal approach involving RNA interference (RNAi) for the targeted depletion of specific components. We focused on the ubiquinol-cytochrome c reductase, Rieske iron–sulfur polypeptide 1 (RISP, also known as UQCRFS1 or complex III, subunit 5), an essential nuclear-encoded subunit of complex III. Disrupting RISP is known to compromise the structure and function of complex III [59]. For the inhibition of complex V, we chose to target ATP5G1, also known as ATP5MC1, which encodes the membrane subunit c of the mitochondrial ATP synthase, a component of the proton channel of complex V.

**Figure 4:**
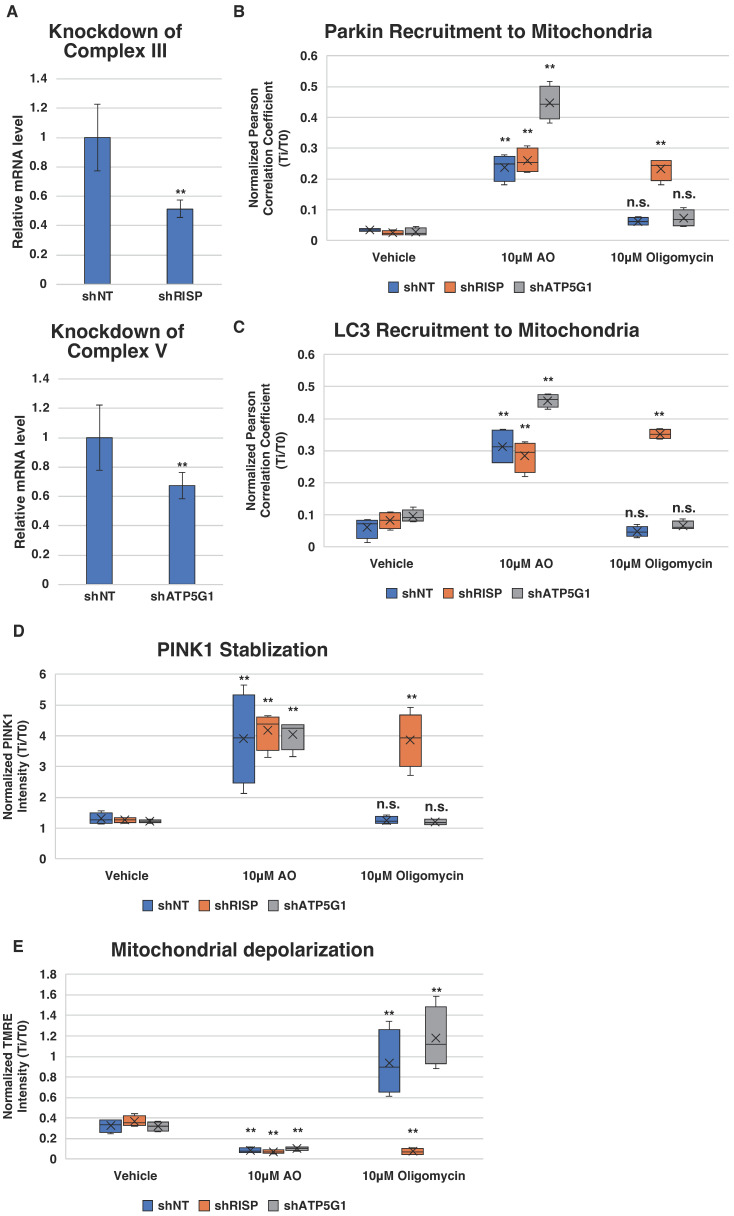
Complex III and complex V knockdowns confirm dual inhibition induced PINK1–Parkin pathway activation. (**A**) RT-qPCR for HeLa complex III and complex V knockdowns. Total RNA of samples was extracted, and RT-qPCR was performed. Data were quantified using 2^−ΔΔCT^ method, and TATA binding protein (TBP) was used as housekeeping gene for quantification (*n* = 4). (**B**–**E**) For each experiment and each condition, four wells of 5000 HeLa cells expressing shNT, shRISP and shATP5G1 were treated with vehicle control, 10 µM of AO or 10 µM of oligomycin, and assays were performed as described previously. Pearson’s colocalization coefficient between RFP-MTS-Smac and Venus-Parkin was measured to quantify colocalization of Parkin to mitochondria using method#3 mentioned in method section. Student’s *t*-test was used for statistical analysis of data (*n* = 4). ** *p* < 0.01 and n.s. > 0.05.

We established HeLa stable cell lines expressing shRNA targeting either RISP (shRISP) or ATP5G1 (shATP5G1), as well as a non-targeting control (shNT). These cell lines also stably expressed either Venus-Parkin, RFP-Smac, and CFP-LC3, or PINK1-EGFP. Quantitative real-time PCR confirmed the successful knockdown of RISP and ATP5G1 (Figure 4A). No Parkin mitochondrial recruitment was observed with the vehicle treatment (Figure 4B). In the presence of 10 µM of AO, both shNT and shRISP cell lines exhibited a similar increase in Parkin mitochondrial translocation, while the ATP5G1 knockdown line showed even more robust Parkin translocation (Figure 4B, middle group). When these cell lines were treated with 10 µM of oligomycin A alone, neither shNT nor shATP5G1 lines showed significant Parkin mitochondrial recruitment, suggesting that oligomycin A alone is insufficient to induce Parkin translocation. However, in shRISP HeLa cells, Parkin translocation was observed upon treatment with oligomycin A in a manner similar to AO treatment (Figure 4B, right group). These findings suggest that inhibiting a key subunit of complex III, in conjunction with complex V inhibition, triggers Parkin mitochondrial recruitment.

Consistent with LC3 recruitment and PINK1 stabilization observed with AO or cloflucarban treatment, shRISP knockdown in combination with oligomycin A treatment yielded results similar to those achieved with AO or cloflucarban (Figure 4C,D). We also assessed Δψm changes in response to vehicle, AO, and oligomycin A treatments. As anticipated, AO completely depolarized the mitochondria compared to the vehicle treatment (Figure 4E). Oligomycin A induced mitochondrial hyperpolarization in shNT cells, likely by blocking proton re-entry via ATP synthase, in accordance with previous reports [60]. A slightly higher hyperpolarization was observed in shATP5G1 cells upon oligomycin A exposure, possibly resulting from a more robust inhibition of the proton channel. Conversely, oligomycin A treatment in shRISP cells led to mitochondrial depolarization, mimicking the combined effect of antimycin and oligomycin A. Taken together, our results further substantiate the hypothesis that the specific inhibition of mitochondrial complexes III and V can activate the PINK1–Parkin pathway. Moreover, they confirm that the cloflucarban-induced activation of the PINK1–Parkin pathway results from its dual inhibitory effects on these complexes.

### 2.5. Bovine Serum Albumin Suppresses Parkin Mitochondrial Translocation Induced by CCCP

**Figure 5:**
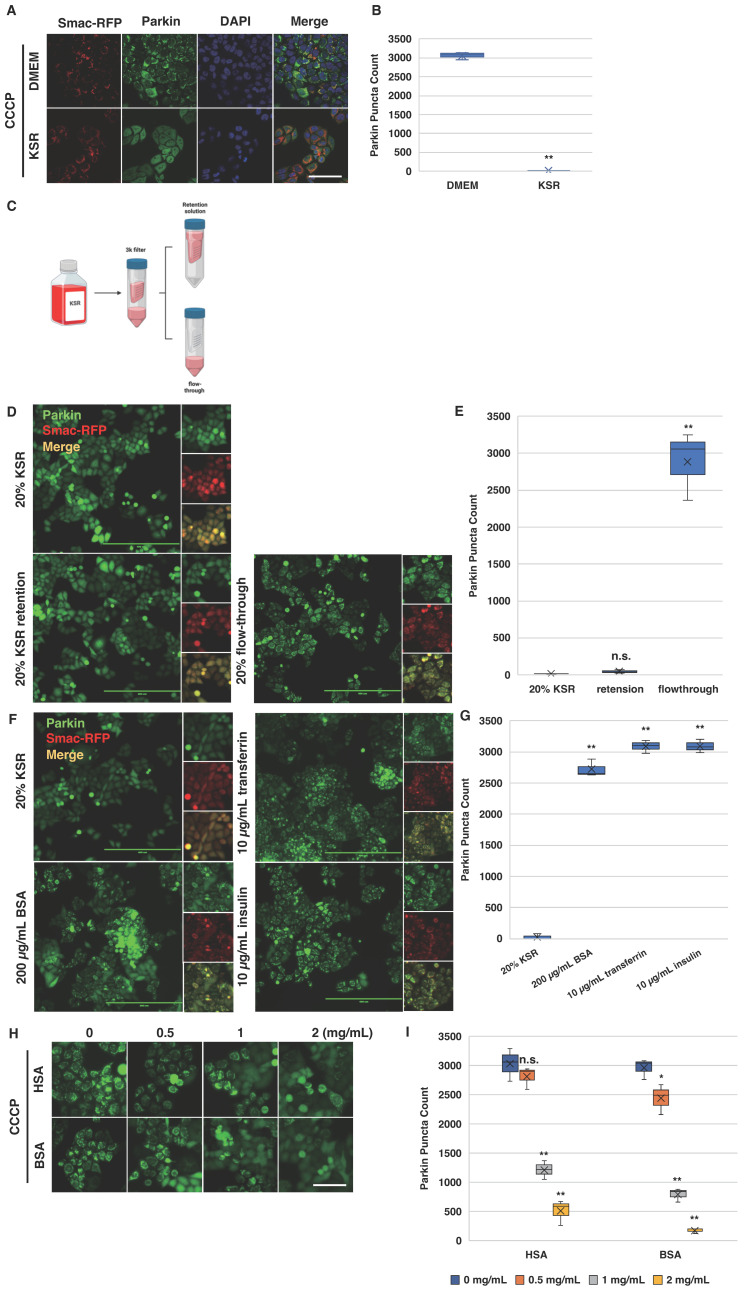
Bovine serum albumin suppresses Parkin mitochondrial translocation induced by CCCP. (**A**,**B**) KSR inhibits CCCP-induced Parkin colocalization in HeLa cells. For each condition, four wells of 5000 HeLa cells expressing Venus-Parkin Smac-RFP were cultured in the indicated medium for 12 h at 37 °C and 5% CO_2_. After incubation, 20 µg/mL Hoechst 33258 was added to each well for 30 min, followed by replacement with 20 µM of CCCP in the indicated medium. Images were captured after a 2 h incubation period. Total numbers of Parkin puncta of cells for each well were collected after the incubation using method#2 mentioned in method section (*n* = 4). Scale bar = 100 µm. (**C**) Schematic of the KSR investigation assay. KSR medium was filtered using a 3 kDa centrifugal filter tube. Both the top and bottom layers were collected for subsequent experiments. (**D**,**E**) Examination of KSR’s inhibition of CCCP-induced Parkin colocalization. Moreover, 20% of KSR, KSR retention, or flow-through was combined with 80% DMEM. Furthermore, 10 µM of CCCP was added to each medium, and for each condition, four wells of 5000 HeLa cells expressing Venus-Parkin and Smac-RFP were treated with the indicated mediums. Images were captured after a 2 h incubation period. Total numbers of Parkin puncta of cells for each well were collected after the incubation using method#2 mentioned in method section (*n* = 4). Scale bar = 400 µm. (**F**,**G**) Investigation of major macromolecules in KSR retention. DMEM was supplemented with 20% KSR, 200 µg/mL BSA, 10 µg/mL transferrin, or 10 µg/mL insulin. Furthermore, 10 µM of CCCP was added to each medium, and for each condition, four wells of 5000 HeLa cells expressing Venus-Parkin and Smac-RFP were treated accordingly. Images were captured after a 2 h incubation period. Total numbers of Parkin puncta of cells for each well were collected after the incubation using method#2 mentioned in method section (*n* = 4). Scale bar = 400 µm. (**H**,**I**) BSA and HSA exhibit similar potencies in inhibiting CCCP-induced Parkin colocalization. For each condition, four wells of 5000 HeLa cells expressing Venus-Parkin were treated with 20 µM of CCCP in DMEM containing either HSA or BSA at the indicated concentrations. Images were captured after a 2 h incubation period. Total numbers of Parkin puncta of cells for each well were collected after the incubation using method#2 mentioned in method section (*n* = 4). Scale bar = 100 µm. * *p* < 0.05, ** *p* < 0.01 and n.s. > 0.05.

Cell culture conditions are known to influence the robustness of Parkin mitochondrial recruitment in response to CCCP treatment. However, the reasons for the variability in Parkin activation via CCCP across different culture media are not fully understood. We observed that when HeLa cells were cultured in KnockOut™ Serum Replacement (KSR)—a medium typically used for embryonic stem cells (ESCs) and induced pluripotent stem cells (iPSCs)—no Parkin mitochondrial translocation occurred upon CCCP treatment. In contrast, the same cell line exhibited a robust response in the DMEM medium (Figure 5A,B).

To investigate whether small or large molecules present in the KSR medium were responsible for this observed effect, we filtered KSR through a 3 kDa centrifugal filter. We collected the top layer as the retention solution and the bottom layer as the flow-through. These two layers were then mixed with 80% standard DMEM, which showed no inhibitory activity in our experiments (Figure 5C). Interestingly, both 20% KSR and its retention solution inhibited CCCP-induced Parkin colocalization, whereas the flow-through did not (Figure 5D,E). This suggests that the inhibitory factor is likely larger than 3 kDa and is probably a protein.

The major protein components of KSR are BSA, transferrin, and insulin. To pinpoint the responsible component, we tested each of these proteins individually in DMEM with a suboptimal concentration of 10 µM of CCCP. At a concentration of 200 µg/mL in DMEM, BSA inhibited CCCP-induced Parkin mitochondrial recruitment, while insulin and transferrin showed no such effect (Figure 5F,G). Given the structural similarity between HSA and BSA, we hypothesized that HSA might also inhibit CCCP’s activity. As expected, both HSA and BSA ranging from concentrations of 1 to 2 mg/mL suppressed 20 µM of CCCP-induced Parkin mitochondrial recruitment in HeLa cells (Figure 5H,I). These findings offer a potential explanation for the inconsistent results observed in studies of CCCP-induced PINK1–Parkin pathway activation. Variations in the composition of culture media, particularly in the concentrations of HSA or BSA, could significantly impact the efficacy of CCCP.

### 2.6. Albumin Modulates Cellular Response to CCCP-Induced Parkin Mitochondrial Translocation

**Figure 6:**
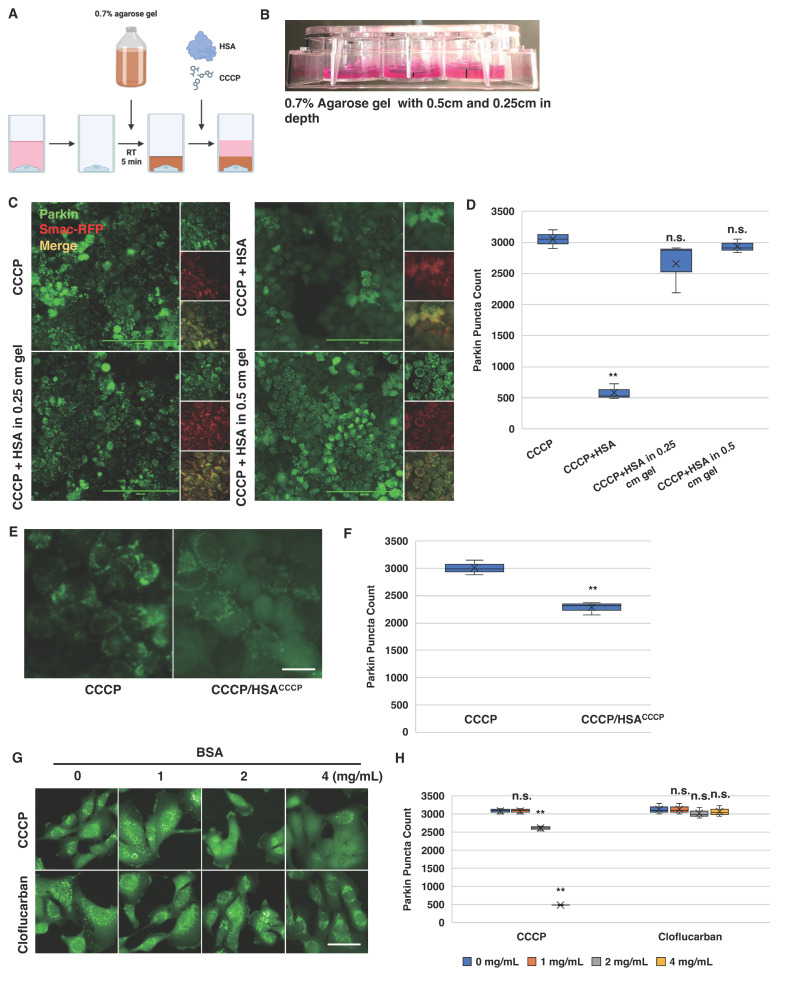
Cloflucarban is a superior activator of Parkin colocalization compared to CCCP in the presence of albumin in the medium. (**A**,**B**) Schematic of the agarose diffusion assay. HeLa cells expressing Venus-Parkin Smac-RFP were seeded into a 12-well plate and incubated for 12 h in DMEM at 37 °C and 5% CO_2_. Following incubation, DMEM was replaced with 42 °C, 0.7% agarose in DMEM. The plate was incubated at room temperature for 5 min until the agarose solidified. Additional DMEM with the specified conditions was added to the wells, and images were captured after a 2 h incubation period and shown in (**C**,**D**). CCCP = 20 µM, and HSA = 2 mg/mL. Total numbers of Parkin puncta of cells for each well were collected after the incubation using method#2 mentioned in method section (*n* = 4). Scale bar = 200 µm. (**E**,**F**) HSA’s inhibition of CCCP-induced Parkin colocalization is not due to direct HSA-CCCP interaction. Moreover, 2 mg/mL HSA was mixed with 20 µM of CCCP in DMEM and incubated for 2 h at 37 °C with constant rocking. After incubation, an additional 10 µM of CCCP was added to the mixture (10 µM of CCCP/HSA^CCCP^), and four wells of 5000 HeLa cells expressing Venus-Parkin were treated with either 10 µM of CCCP or 10 µM of CCCP/HSA^CCCP^. Images were captured after a 2 h incubation period. Total numbers of Parkin puncta of cells for each well were collected after the incubation using method#2 mentioned in method section (*n* = 4). Scale bar = 100 µm. (**G**,**H**) Cloflucarban but not CCCP activates Parkin colocalization in the presence of BSA. Four wells of 5000 MEF cells expressing Venus-Parkin were treated with either 20 µM of CCCP or 20 µM of cloflucarban in the presence of BSA at the indicated concentrations. Images were captured after a 2 h incubation period. Total numbers of Parkin puncta of cells for each well were collected after the incubation using method#2 mentioned in method section (*n* = 4). Scale bar = 50 µm. ** *p* < 0.01 and n.s. > 0.05.

Considering that both BSA and HSA are known to directly bind small molecules, one potential explanation for their inhibitory effects on CCCP is that BSA/HSA could sequester CCCP, thereby preventing its cellular uptake and neutralizing its mitochondrial targeting. Alternatively, BSA or HSA might directly influence cell behavior, rendering them less responsive to CCCP. To differentiate between these mechanisms, we employed an agarose diffusion assay [61,62,63,64]. The premise was that an agarose gel layer placed atop the cells would prevent direct interactions between BSA or HSA in the medium and the cells (Figure 6A). If BSA/HSA sequesters CCCP, the equilibrium would shift in favor of their association in the medium, thereby inhibiting CCCP diffusion through the agarose gel (Figure 6A). In brief, HeLa cells expressing Venus-Parkin and Smac-MTS-RFP were seeded into 12-well plates and incubated overnight. Once the cells adhered to the plate, DMEM was replaced with 0.7% agarose in DMEM pre-warmed to 42 °C and allowed to gel at room temperature for 5 min. Subsequently, fresh DMEM was added to each well under the indicated conditions, and images were captured following 2 h of treatment. As shown in Figure 6B–D, Parkin mitochondrial recruitment occurred in HeLa cells treated with both 0.25 cm and 0.5 cm agarose gels. This finding contradicts the notion of CCCP neutralization by direct BSA or HSA binding but supports the idea that HSA modulates cellular response to CCCP.

As an independent confirmation of this HSA-mediated mechanism, we carried out an HSA saturation assay. Briefly, 2 mg/mL of HSA was mixed with 20 µM of CCCP in DMEM and incubated at 37 °C for 2 h with constant mixing. After incubation, an additional 10 µM of CCCP was introduced to the mixture and applied to HeLa cells expressing Venus-Parkin. Cells treated with 10 µM of CCCP alone served as controls. Under these conditions, if HSA-CCCP interaction neutralizes CCCP activity, an extra dose of CCCP should induce Parkin mitochondrial recruitment. However, the HSA saturation experiment revealed that cells exposed to HSA remained unresponsive to additional CCCP doses, implying that HSA exposure hinders the activation of the PINK1–Parkin pathway via CCCP (Figure 6E,F).

### 2.7. Cloflucarban Induces Parkin Mitochondrial Translocation Independent of BSA

As cloflucarban prompts Parkin mitochondrial recruitment through the dual inhibition of mitochondrial complexes III and V, a mechanism distinct from that of CCCP, we investigated whether HSA or BSA also affect cloflucarban-induced Parkin translocation. To establish the broader applicability of BSA’s inhibitory effect on either CCCP- or cloflucarban-induced Parkin translocation, we utilized MEF cells stably expressing Venus-Parkin. Similar to HeLa cells, CCCP-induced Parkin translocation was almost entirely suppressed by 4 mg/mL of BSA (Figure 6G,H). In contrast, cloflucarban-induced Parkin translocation remained unaffected by BSA at all tested concentrations. This finding is consistent with our hypothesis that cloflucarban activates the PINK1–Parkin pathway via a mechanism distinct from CCCP. Given that Parkin induction by cloflucarban is less sensitive to BSA interference, cloflucarban emerges as a superior or, at the very least, an alternative tool for investigating PINK1–Parkin activation across different cell lines and culture media.

## 3. Discussion

Here, we profiled the mitochondrial complex activity inhibition caused by several small molecules known to affect mitochondrial health and determined whether these small molecules can activate the PINK1–Parkin pathway. We found a strong correlation between Parkin activation and the dual inhibition of complex III and complex V. Of these compounds, cloflucarban possessed this profile and robustly activated Parkin mitochondrial translocation with an EC_50_ < 10 μM in HeLa cells and <4 μM in RPE cells (Supplemental Figures S2 and S3). To further support that the dual inhibition of complex III and complex V is causal for Parkin activation, we showed that knocking down a key component of complex III sensitized cells to Parkin recruitment with oligomycin A treatment, a compound that is insufficient by itself to cause Parkin recruitment. These results provide further support for the hypothesis that PINK1/Parkin actively monitors mitochondrial complex health and function under physiological conditions. Finally, we explored the root cause of inconsistent PINK1–Parkin pathway activation across different culture conditions and identified that albumin in the cell culture media diminishes the effectiveness of CCCP-induced Parkin mitochondrial translocation. In contrast, cloflucarban activates the PINK1–Parkin pathway independently of albumin. Taken together, our research suggests that cloflucarban can be an alternative, physiologically relevant, and effective tool for the investigation of Parkin-dependent mitophagy in vitro.

Previous studies have revealed several methods to trigger PINK1–Parkin pathway-induced mitophagy, including the expression of misfolded proteins or the inhibition of chaperones in the mitochondrial matrix, the disruption of mtDNA replication and the depletion induced by the overexpression of mutated Twinkle, and compounds that induce mitochondrial damage [34,35,36,37,38]. Although it is generally considered that mitophagy pathways are activated upon mitochondrial damage, our data and results from other studies [33] indicate that not all mitochondrial-damaging compounds are capable of activating Parkin or Parkin-dependent mitophagy, as demonstrated by myxothiazol, a potent complex III inhibitor (Figure 1). The precise explanation for this phenomenon remains a subject of ongoing investigation. Myxothiazole is known to possess a wide range of cellular activities beyond ETC complex inhibition, which adds to the complexity of understanding its effects on Parkin recruitment. Previous studies have demonstrated that myxothiazole, unlike antimycin A, does not trigger superoxide production and has no discernible impact on autophagy or mitophagy [65,66,67]. Additionally, it has been reported that myxothiazole inhibits superoxide production via complex III [68]. Notably, superoxide or reactive oxygen species have been implicated as necessary factors for inducing the mitochondrial recruitment of Parkin [69,70]. Thus, one plausible explanation for myxothiazole’s inability to induce Parkin mitochondrial recruitment could be its failure to stimulate ROS production in our experimental cells. Furthermore, myxothiazole has been shown to induce the phosphorylation of translation initiation factor eIF2α, thereby inhibiting global translation initiation and promoting the integrated stress response (ISR) [71]. In a previous study, we showed that PINK1 is an unstable protein and the elevation of PINK1 in response to CCCP requires a robust PINK1 translation [41]. Therefore, an alternative explanation for myxothiazole’s failure to trigger Parkin mitochondrial recruitment could be linked to its suppression of PINK1 translation.

It appears that the PINK1–Parkin pathway monitors specific mitochondrial dysfunctions to induce mitophagy responses. Although our previous results showed sorafenib inhibits both complex III and complex V of the ETC and activates the PINK1–Parkin pathway [72], there are significant uncertainties about whether the dual inhibition of complex III and complex V is sufficient to induce Parkin activation since sorafenib is primarily a multi-kinase inhibitor. In this study, we first demonstrated that a complex III and V inhibitor, cloflucarban, is a potent activator of this pathway. Additionally, we showed that knocking down a key subunit of complex III with shRNA phenocopies the complex III inhibitor in combination with chemical complex V inhibition in activating this pathway. Collectively, these new results further strengthen the notion that the dysfunction of complexes III and V may be one of the endogenous signals that activate the PINK1–Parkin pathway. Since previous studies have shown Parkin or PINK1 knockout mice accumulate mtDNA mutations following exhaustive exercise and induce STING-dependent innate immune responses [39], it will be interesting to test, in the future, if the disruption of complex III and complex V elicits mitochondrial DNA damage to activate PINK1/Parkin.

The intriguing observation that CCCP failed to induce Parkin colocalization in HeLa cells cultured with the KSR media underscores the importance of understanding the cellular microenvironment when studying molecular pathways. The differential activation of the PINK1–Parkin pathway between various cell types, as noted in previous studies, suggests that external factors, such as culture medium components, can significantly influence cellular responses [32]. Our findings that both BSA and its human counterpart, HSA, inhibit CCCP-induced Parkin colocalization highlight the potential pitfalls of experimental setups. Our result is consistent with previous observations that FBA/BSA media concentration influences CCCP’s capability to activate PINK1–Parkin mitophagy [73,74]. One possible mechanism for negating CCCP activity has been attributed to the binding of CCCP to BSA, reducing the effective concentration of CCCP; however, other mechanisms could also be in play since simply increasing CCCP concentration in the media cannot overcome the BSA effect. It should be noted that CCCP has been shown to have other biological activities other than mitochondrial depolarization. For example, Ramm and coworkers showed that CCCP interferes with lysosomal function and autophagosomal degradation in both yeast and mammalian cells [75]. Others showed that CCCP can equilibrate the pH of lysosomes [76], the Golgi complex [77,78] and microtubules [79]. Hence, CCCP-induced PINK1/Parkin activation may not be a result of mitochondrial depolarization alone. The complexity of CCCP’s mechanism of action in activating PINK1/Parkin underscores the need to develop orthogonal tools to investigate the upstream mechanism of PINK1/Parkin activation. In this regard, cloflucarban offers an alternative and potentially more reliable tool for studying this pathway since cloflucarban is insensitive to BSA or HSA in activating this pathway, reducing one possible variability from cell culturing conditions.

In conclusion, our study provides additional evidence that the dual inhibition of complexes III and V is sufficient to activate the PINK1–Parkin pathway, suggesting dysfunctional complexes III and V could be one of the endogenous signals for activating mitophagy. The discovery of cloflucarban as a strong activator of PINK1/Parkin further underscores the importance of this mechanism. Furthermore, our finding that the activation of this pathway by cloflucarban, but not CCCP, is insensitive to BSA or HSA interference highlights cloflucarban’s potential as a more consistent and reliable tool for inducing PINK1/Parkin-dependent mitophagy and the need for using orthogonal tools to elucidate a signaling pathway.

## 4. Methods and Materials

### 4.1. Mitochondrial Complex Activity Assays

The MitoTox Complete OXPHOS Activity Assay Kit (Abcam, Waltham, MA, USA, ab110419) was used to assess the inhibition of mitochondrial complex I-V activity by the compounds of interest. Each complex’s activity was measured individually following the manufacturer’s instructions. Briefly, to evaluate complex I, II, IV and V activity, bovine heart mitochondria were solubilized, and 15 µg of mitochondrial solution was added to each well in a 96-well plate. These plates were precoated with antibodies to pull down specific complexes. For the complex I assessment, phospholipids were added to wells, and then the vehicle, 10 µM of rotenone, or 50 µM of a specified compound were added along with complex I activity solution that included ubiquinone 1 and NADH. The activity was quantified as the decrease in NADH 340 nm absorbance at room temperature in kinetic mode every 2 min for 1 h. For the complex II assessment, the vehicle, 10 µM of rotenone, or 50 µM of a specified compound was combined with complex II activity solution containing ubiquinone, DCPIP and succinate, and this was added to the 96-well precoated plate. The activity was quantified by monitoring the decrease in DCPIP 600 nm absorbance at room temperature in kinetic mode every 2 min for 1 h. For the complex IV evaluation, the vehicle, 10 µM of rotenone, or 50 µM of a specified compound was combined with complex IV activity solution containing cytochrome c, and this was added to the 96-well precoated plate. The activity was quantified by monitoring the decrease in cytochrome c 550 nm absorbance caused by oxidation at room temperature in kinetic mode every 2 min for 1 h. For the complex V assessment, phospholipids were added to the 96-well precoated plates. After a 45 min incubation period, the vehicle, 10 µM rotenone, or 50 µM of a specified compound was combined with complex V activity solution containing phosphoenolpyruvate, pyruvate kinase, lactate dehydrogenase, and NADH, which was added to the 96-well plate. ATP production from ATP synthase was coupled to NADH oxidation with the contents of the complex V activity solution. Thus, the activity was assessed by monitoring the decrease in NADH 340 nm absorbance caused by oxidation at room temperature in kinetic mode every 2 min for 1 h. For complex II/III evaluations, the vehicle, 10 M rotenone, or 50µM of a specified compound was combined with complex III activity solution containing succinate, cytochrome c, KCN, and rotenone, and then these were added to the 96-well plates. Bovine heart mitochondria were prepared and added to the 96-well plates and mixed. The activity was determined by measuring the increase in reduced cytochrome C 550 nm absorbance caused by oxidation at room temperature in kinetic mode every 2 min for 1 h. All MitoTox data were collected on the Tecan Safire reader. Custom MATLAB scripts were used to analyze the rate of absorbance change for all the assays and to generate graphs.

### 4.2. Cell Culture, Transfection, and Reagent Treatment

HEK293T cells were obtained from ATCC. HeLa cells were a gifted by Dr. Sabrina Spencer. Parkin and PINK1 knockout MEF cells were obtained from Dr. Jie Shen and described previously [17]. Beige fat cells were a gift from Dr. Shingo Kajimura. The cells were maintained in Dulbecco’s Modified Eagle’s Medium (DMEM) supplemented with 10% fetal calf serum (Invitrogen, Carlsbad, CA, USA), penicillin, streptomycin (100 units/mL), and 1 mM of L-glutamine. All the stable cell lines made by lentivirus packaging were selected with 100 μg/mL of hygromycin (Alexis Biochemicals, Farmingdale, NY, USA), 5 μg/mL of blasticidin (Invitrogen, Carlsbad, CA, USA), or 2 μg/mL of puromycin (Sigma Aldrich, Burlington, MA, USA) based on the selection markers. The cells were treated at indicated concentrations with CCCP, myxothiazole and antimycin (Sigma Aldrich, Burlington, MA, US), oligomycin, nicardipine, trans-resveratrol, ciglitazone, simvastatin, troglitazone, and 1-NA-PP1 (Cayman Chemical, Ann Arbor, MI, USA), and cloflucarban (Alfa Aesar, Ward Hill, MA, USA).

### 4.3. Live Cell Imaging and Fluorescence Microscopy

In the initial complex inhibitor experiments, Parkin recruitment assays, cells were grown in Costar 3603 96-well plates (Corning, Corning, NY, USA) at 5000 cells per well at 37 °C and 5% CO_2_ in a humidified incubator. The cells were imaged at 5% CO_2_ and 37 °C in a ImageXpress XL system (Molecular Devices, San Jose, CA, USA) with the laser and filter cubes for YFP and mCherry using a 10× air NA 1.1 objective. Live cell images were collected for 0–2 h post treatment.

For TMRE assays, HeLa cells were grown in Costar 3603 96-well plates (Corning, Corning, NY, USA) at 5000 cells per well at 37 °C and 5% CO_2_ in a humidified incubator. The cells were imaged at 5% CO_2_ and 37 °C in a ImageXpress XL system (Molecular Devices, San Jose, CA, USA) with the laser and filter cubes for YFP and mCherry using a 10× air NA 1.1 objective. Live cell images were collected for at a time point before treatment and 0–2 h post treatment.

For cloflucarban characterization experiments, Parkin recruitment and mitophagy assays, cells were grown in Cell Carrier Ultra 96-well plates (Perkin Elmer, Waltham, MA, USA) at 5000 cells per well at 37 °C and 5% CO_2_ in a humidified incubator. The cells were imaged at 5% CO_2_ and 37 °C in a Opera Phenix High-Content Screening System (Perkin Elmer, Waltham, MA, USA) with the laser and filter settings for CFP, YFP, and mCherry using a 40× water NA 1.1 objective. Live cell images were collected for 0–22 h post treatment.

For PINK1 stabilization and mitochondrial membrane potential assays, 5000 cells per well were grown in Cell Carrier Ultra 96-well plates (Perkin Elmer, Waltham, MA, USA) at 37 °C and 5% CO_2_ in a humidified incubator.

The cells were imaged at 5% CO_2_ and 37 °C in an Opera Phenix High-Content Screening System (Perkin Elmer, Waltham, MA, USA) with the laser and filter settings for EGFP and TRITC using a 20× air NA 0.8 objective. Live cell images were collected for a pre-treatment time point and 0–8 h after treatment.

### 4.4. Mitochondrial Membrane Potential Assay and Quantification

Mitochondria were stained with TMRE dye (Thermo Fisher, Waltham, MA, USA) as described previously [72]. The cells were imaged for 20 ms at 20% power using the TRITC filter on the Opera Phenix (Perkin Elmer, Waltham, MA, USA). The membrane potential was quantified by using MATLAB (Mathworks, Natick, MA, USA) to background-subtract and threshold images. The average TRITC pixel intensity was calculated for each image. For each time point, the values were normalized to the pre-treatment value for the corresponding site.

### 4.5. Parkin Mitochondrial Recruitment Assays

Three methods were employed to quantify Parkin mitochondrial translocation, each adapted to the specific microscopy platform utilized for data acquisition. These methods are the following: Method 1—Parkin puncta formation using the ImageXpress (Molecular Devices, San Jose, CA, USA), Method 2—Parkin puncta formation using the Opera Phenix (Perkin Elmer, Waltham, MA, USA), and Method 3—Parkin colocalization with mitochondria. In Method 1, Venus-Parkin and RFP-Smac (a mitochondrial marker) were imaged with the ImageXpress (Molecular Devices, San Jose, CA, USA). The resultant images were then analyzed using the MetaXpress (Molecular Devices, San Jose, CA, USA) analysis suite. This analysis involved quantifying YFP puncta, applying an intensity threshold of 800 and setting a minimum puncta size of 2 pixels. Method 2 involved imaging Venus-Parkin with the Opera Phenix (Perkin Elmer, Waltham, MA, USA), followed by image quantification using the Harmony analysis suite. YFP puncta counts for each image were recorded using Method B in the suite, with a local threshold set at 0.098. For Method 3, Venus-Parkin and RFP-Smac images were processed in MATLAB (Mathworks, Natick, MA, USA). This process included background subtraction and thresholding, followed by the calculation of Pearson’s correlation coefficient for each site. These coefficients were then averaged across wells and replicates. All three methods demonstrated consistent results in assessing the efficiency of Parkin mitochondrial recruitment.

### 4.6. Mitophagy Assays

Mitophagy was quantified using co-localization experiments between LC3 and mitochondria (RFP-Smac). MATLAB (Mathworks, Natick, MA, USA) was used to process images using background subtraction and thresholding before Pearson’s correlation coefficient was calculated for each site and averaged across wells and replicates as described previously [17,72].

### 4.7. PINK1 Stabilization Assay

PINK1-EGFP stabilization was quantified using MATLAB (Mathworks, Natick, MA, USA) to background-subtract and threshold images. The average EGFP pixel intensity was calculated for each image. For each time point, the values were normalized to the pre-treatment value for the corresponding site.

### 4.8. Mitochondria Superoxide Production Assay

Mitochondria were stained with MitoSox dye (Thermo Fisher, Waltham, MA, USA) following the protocols provided by the manufacturer. The cells were imaged for 100 ms at 40% power using the TRITC filter on the Opera Phenix (Perkin Elmer, Waltham, MA, USA). Membrane potential was quantified by using MATLAB to background-subtract and threshold images. The average TRITC pixel intensity was calculated for each image. For each time point, the values were normalized to the pre-treatment value for the corresponding site.

### 4.9. Parkin Mitochondrial Recruitment Assay with PINK1-M318A Activity Inhibition

PINK1 and Parkin double-knockout MEF cells were transduced with human PINK1-M318A and Venus-Parkin as described previously [80]. After being seeded, these cells were treated with 5 µM of 1-NA-PP1 for 20 min, which inhibits PINK1 phosphorylation activity. The cells were then treated with cloflucarban, AO and the vehicle as indicated and imaged for Venus-Parkin expression. The images were quantified using the Harmony analysis suite (Perkin Elmer, Waltham, MA, USA). The count of YFP puncta was then recorded for each image using Method 2 in the analysis suite with a 0.098 local threshold.

### 4.10. shRNA Knockdown and Lentiviral Transduction

Lentiviral vectors expressing shRNA targeting RISP (TRCN0000046518) and ATP5G1 (TRCN0000043484) were purchased from Sigma-Aldrich (Burlington, MA, USA). The non-targeting lentiviral vector used was SHC002 (Sigma, Burlington, MA, USA, Cat. No SHC002) which contains shRNA with no homology to either human or mouse targets. HEK293T cells were plated to 60–80% confluency one day before transfection. For each transfection, a mixture of 500 μL warm media, 40 μL PEI at 45 °C and 1.5 μg of the plasmids VSV-G, RSV-Rev and Pmdl were made in 1.5 mL tubes. Furthermore, 3 μg of target DNA (NT, RISP, or ATP5G1) was added to the specific tube, gently mixed, and incubated for 15 min. The content of each tube was added to 6 cm dishes with the HEK293T cells, and they were incubated over night at 37 °C and 5% CO_2_. Moreover, 3 mL of the media of the transfected cells were taken and added to the plate of cells that were to be transfected using a 0.2 μm filter. Furthermore, 3 mL of fresh media was added to the HEK293T transfected cells afterwards, and all plates were incubated in the viral incubator for two days. Transfected cells with high confluency were split before they were transfected again with 3 mL of viral media of HEK293T plates using a 0.2 μm filter. The transfected cells were split to low confluency, and puromycin (1 μg/mL) was added for selection. The selection with puromycin was repeated before the cells were used for further analysis.

### 4.11. RNA Extraction and Quantitative Real-Time PCR

Total RNA was isolated from the samples using TRIzol reagent (Invitrogen, Carlsbad, CA, USA) according to the manufacturer’s protocol. The samples were homogenized in 1 mL of TRIzol reagent and incubated at room temperature for 5 min. 200 µL Chloroform (Thermo Fisher Scientific, Waltham, MA, USA) was added to the homogenate, shaken vigorously for 15 s, and allowed to stand at room temperature for 2–3 min. The mixture was then centrifuged at 12,000× *g* for 15 min at 4 °C. The aqueous phase was transferred to a fresh tube, and RNA was precipitated by adding an equal volume of isopropanol. After 10 min of incubation at room temperature, the samples were centrifuged at 12,000× *g* for 10 min at 4 °C. The RNA pellet was washed with 75% ethanol, air-dried, and resuspended in RNase-free water. The concentration and purity of the RNA were determined using a NanoDrop spectrophotometer (Thermo Fisher Scientific, Waltham, MA, USA).

The extracted RNA was reverse-transcribed into complementary DNA (cDNA) using a suitable reverse transcription kit (Thermo Fisher Scientific, Waltham, MA, USA). The reaction was performed according to the manufacturer’s instructions, typically including a primer annealing step, a reverse transcription step, and a termination step. The resulting cDNA was stored at −20 °C until further use.

RT-qPCR was performed using the Luna^®^ Universal qPCR Master Mix (New England Biolabs, Ipswich, MA, USA) according to the manufacturer’s instructions. Briefly, the 20 µL reaction volume consisted of 10 µL of Luna Universal qPCR Master Mix, 1 µL of each forward and reverse primer (10 µM), 2 µL of template cDNA, and 6 µL of nuclease-free water. The reactions were run on a real-time PCR system (Applied Biosystems, Foster City, CA, USA) with the following thermal cycling conditions: initial denaturation at 95 °C for 1 min, followed by 40 cycles of denaturation at 95 °C for 15 s, annealing at 60 °C for 30 s, and extension at 72 °C for 30 s. The expression of the target genes was normalized to the expression of the housekeeping gene TBP, and the relative gene expression was calculated using the 2^−ΔΔCT^ method. Each sample was run in triplicate, and a no-template control was included to ensure specificity and the absence of contamination.

Primers used for qRT-PCR are as follows:

RISP

Forward: 5′-CTGAATACCGCCGCCTTGAA-3′

Reverse: 5′-ATGCGACACCCACAGTAGTTA-3′

ATP5G1

Forward: 5′-CCAGGAACCCGTCTCTCAAG-3′

Reverse: 5′-GGAAGGCGACCATCAAACAGA-3′

TBP

Forward: 5′-TTCGGAGAGTTCTGGGATTGTA-3′

Reverse: 5′-TGGACTGTTCTTCACTCTTGGC-3′

KSR characterization assay

KnockOut™ Serum Replacement (Thermo Fisher Scientific, Waltham, MA, USA) was filtered using a 3 kDa 15 mL centrifugal tube (Amicon Ultra-15, MilliporeSigma, Burlington, MA, USA) in a fixed-angle centrifuge set at 4000× *g* at 4 °C for 30 min (Sorvel RC-5B, rotor F15S-8x50C, Newtown, CT, USA). The original KSR, top, or bottom layers were mixed at 20% with 80% DMEM as described in the cell culture section. HeLa cells expressing Venus-Parkin Smac-RFP were treated under each indicated condition using 20 µM of CCCP as an activator. The Parkin recruitment assay was then performed.

For the retention solution characterization, 20% original KSR, 200 µg/mL bovine serum albumin (BSA, Sigma Aldrich, Burlington, MA, USA), 10 µg/mL transferrin (Roche, Indianapolis, IN, USA), or 10 µg/mL insulin (Sigma Aldrich, Burlington, MA, USA) was mixed with DMEM. Subsequently, the Parkin recruitment assay was conducted for each condition.

For the BSA/HSA evaluation, both BSA and human serum albumin (HSA, Sigma Aldrich, Burlington, MA, USA) were added to DMEM at the indicated concentrations, combined with 20 µM of CCCP. HeLa cells expressing Venus-Parkin Smac-RFP were treated under each specified condition, followed by the Parkin recruitment assay.

### 4.12. Agarose Diffusion Assay

HeLa cells expressing Venus-Parkin Smac-RFP (2 × 10^5^) were seeded into a 12-well plate (Corning, Corning, NY, USA). After incubating for 12 h at 37 °C and 5% CO_2_ in a humidified incubator, DMEM was replaced with 42 °C, 0.7% agarose (Invitrogen, Carlsbad, CA, USA) in DMEM and allowed to set for 5 min at room temperature. Once the agarose solidified, 1 mL of fresh DMEM containing the indicated condition (20 µM of CCCP, 2 mg/mL of HSA) was added to each well. The Parkin recruitment assay was subsequently performed.

### 4.13. HSA Saturation Assay

HSA (2 mg/mL) was combined with 20 µM of CCCP in DMEM and incubated at 37 °C for 2 h with constant rocking (Labquake shaker, Thermo Fisher Scientific, Waltham, MA, USA). Post incubation, an additional 10 µM of CCCP was introduced to the mixture. HeLa cells expressing Venus-Parkin were treated with either 10 µM of CCCP or the mixture, followed by the Parkin recruitment assay.

### 4.14. Cloflucarban Evaluation Assay

BSA was introduced into DMEM at the specified concentrations, combined with either 20 µM of CCCP or 20 µM of cloflucarban. MEF cells expressing Venus-Parkin were treated under each indicated condition, and the Parkin recruitment assay was then conducted.

### 4.15. Statistical Analysis

Statistical significance was determined via Microsoft^®^ Excel^®^ ver. 16.81. Data are represented as mean ± SD. Student’s *t*-test was used for statistical analysis of the data, and a *p*-value < 0.05 was considered statistically significant.

## Data Availability

Material and data are available upon request.

## Supplementary Materials

The following supporting information can be downloaded at: https://www.mdpi.com/article/10.3390/biom14030248/s1, Supplemental Figure S1. Parkin puncta formation in HeLa cells under different drug treatments. Representative images of Parkin localization after treated with indicated conditions in Figure 1G. Cells are labeled with Parkin-YFP, and scale is 50 μm. Supplemental Figure S2. Parkin puncta in HeLa cells 1 h after drug treatment. (A) Representative images of Parkin localization, cells are labeled with Parkin-YFP, and scale is 200 μm. (B) Quantification of Parkin puncta, paired *t*-test of one experiment as triplicate analyzed in GraphPad Prism, error bars show SEM. (C) Concentration series of cloflucarban shows an EC_50_ of 8.5 μM after 1 h based on normalized Parkin puncta count of three independent experiments analyzed with MATLAB using method#2 mentioned in method section. Supplemental Figure S3. Parkin puncta in RPE1 cells 2 h after drug treatment. (A) Representative images of Parkin localization, cells are labeled with Parkin-YFP, scale is 200 μm. (B) Quantification of Parkin puncta, paired *t*-test was used for analysis of three independent experiments, error bars show SEM. (C) Quantification of Parkin puncta in cells treated with concentration series of cloflucarban. (D) Concentration series of cloflucarban shows an EC_50_ of 3.9 μM 2 h after treatment, analysis was performed with MATLAB using the normalized Parkin puncta count of three independent experiments using method#2 mentioned in method section.
