# Supplementary material for: Cloflucarban Illuminates Specificity and Context-Dependent Activation of the PINK1–Parkin Pathway by Mitochondrial Complex Inhibition
Source: Biomolecules. 2024 Feb 20;14(3):248. doi: 10.3390/biom14030248 (PMC10967832; doi:10.3390/biom14030248)
Supplement: Supplementary file 1 [file biomolecules-14-00248-s001.zip › Supplemental Figure S3.pdf]

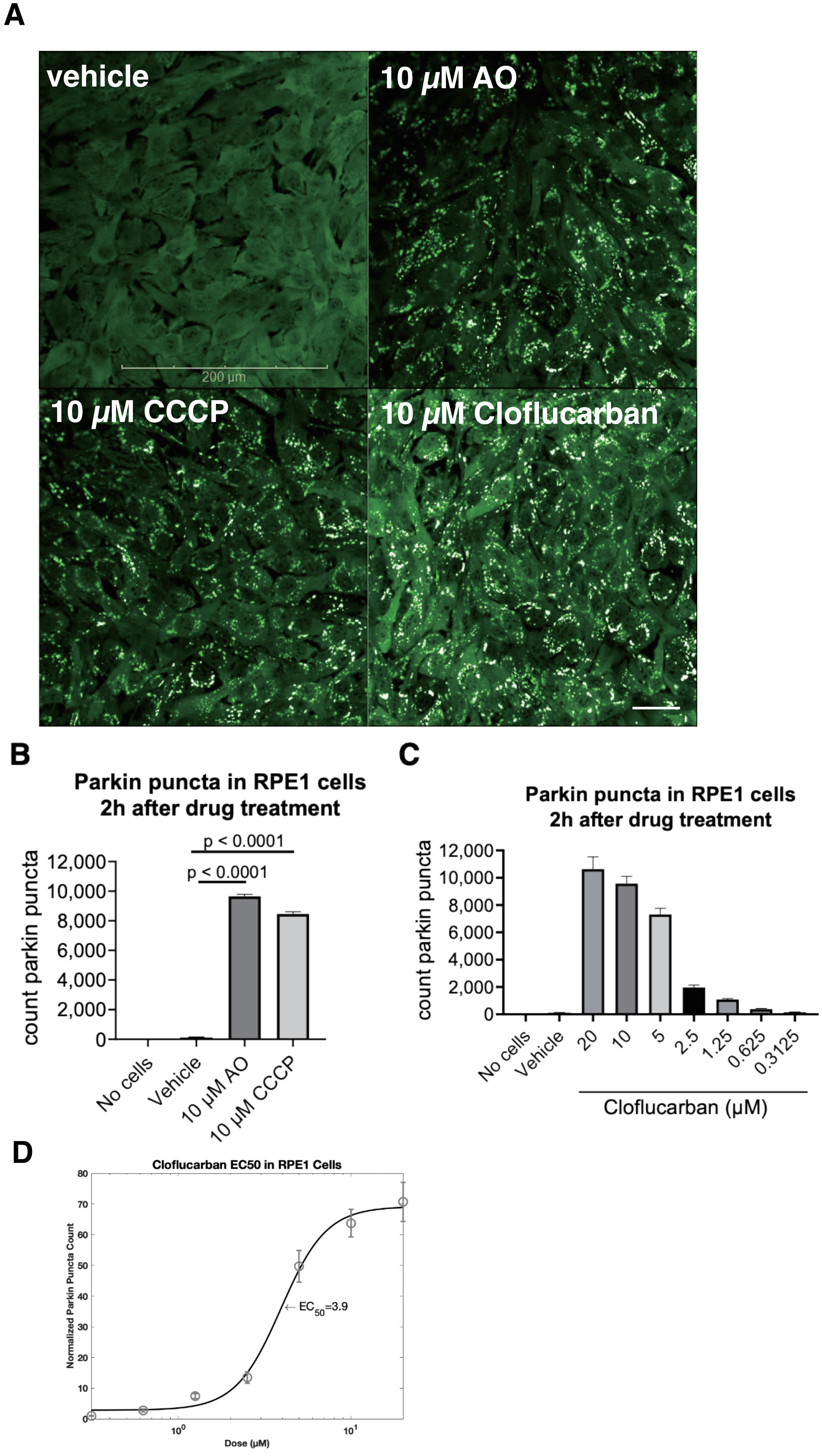
A
vehicle
10 μM AO
10 μM CCCP
10 μM Cloflucarban
200 µm
B
Parkin puncta in RPE1 cells
2h after drug treatment
p < 0.0001
p < 0.0001
count parkin puncta
12,000
10,000
8,000
6,000
4,000
2,000
0
No cells
Vehicle
10 μM AO
10 μM CCCP
C
Parkin puncta in RPE1 cells
2h after drug treatment
count parkin puncta
12,000
10,000
8,000
6,000
4,000
2,000
0
No cells
Vehicle
20
10
5
2.5
1.25
0.625
0.3125
Cloflucarban (μM)
D
Cloflucarban EC50 in RPE1 Cells
Normalized Parkin Puncta Count
80
70
60
50
40
30
20
10
0
EC50=3.9
10^0
10^1
Dose (μM)
