# Supplementary figures and images for: Cloflucarban Illuminates Specificity and Context-Dependent Activation of the PINK1–Parkin Pathway by Mitochondrial Complex Inhibition
Source: Biomolecules. 2024 Feb 20;14(3):248. doi: 10.3390/biom14030248 (PMC10967832; doi:10.3390/biom14030248)

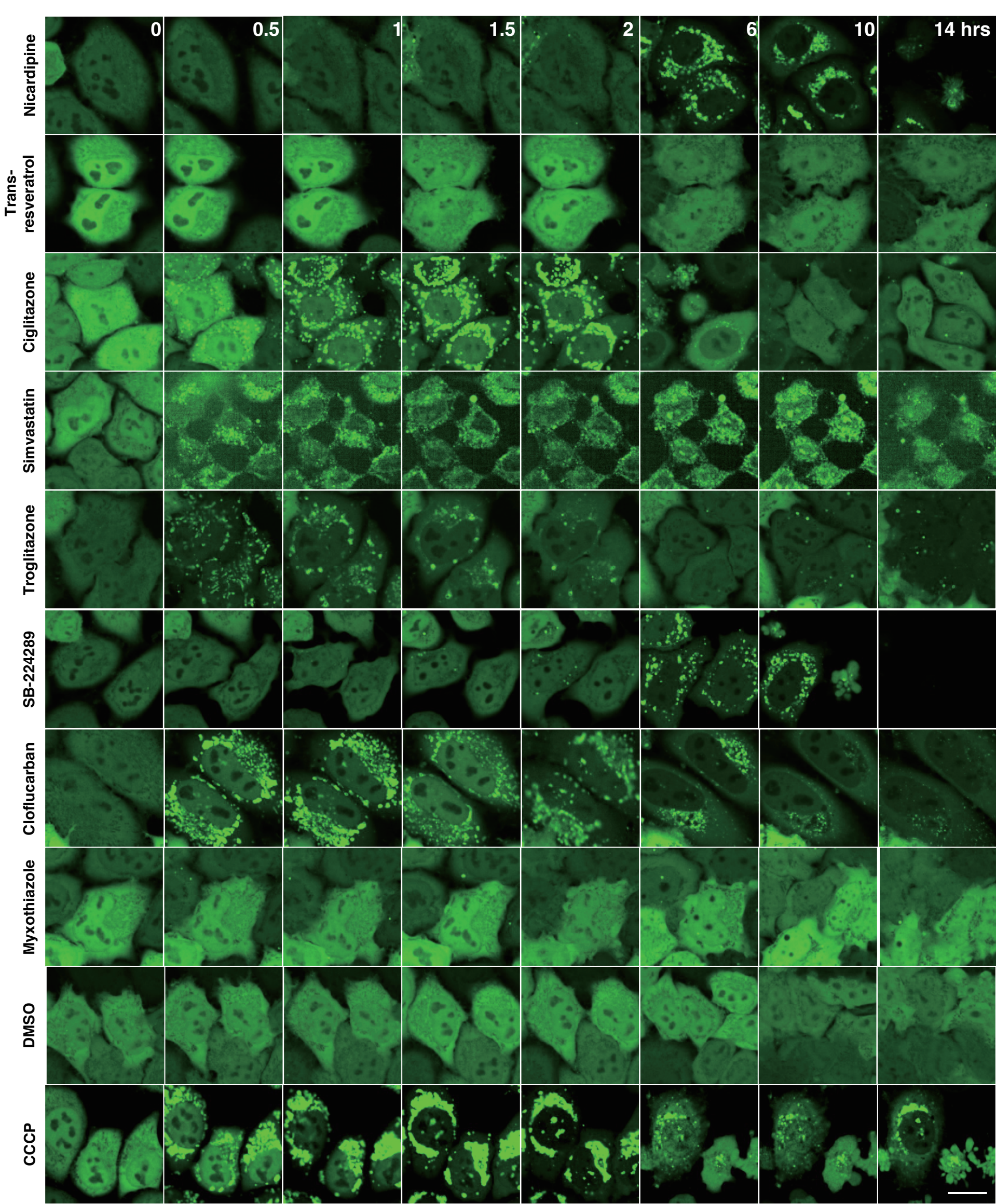

Supplement: Supplementary file 1 [file biomolecules-14-00248-s001.zip › Supplemental Figure S1.pdf]

**A**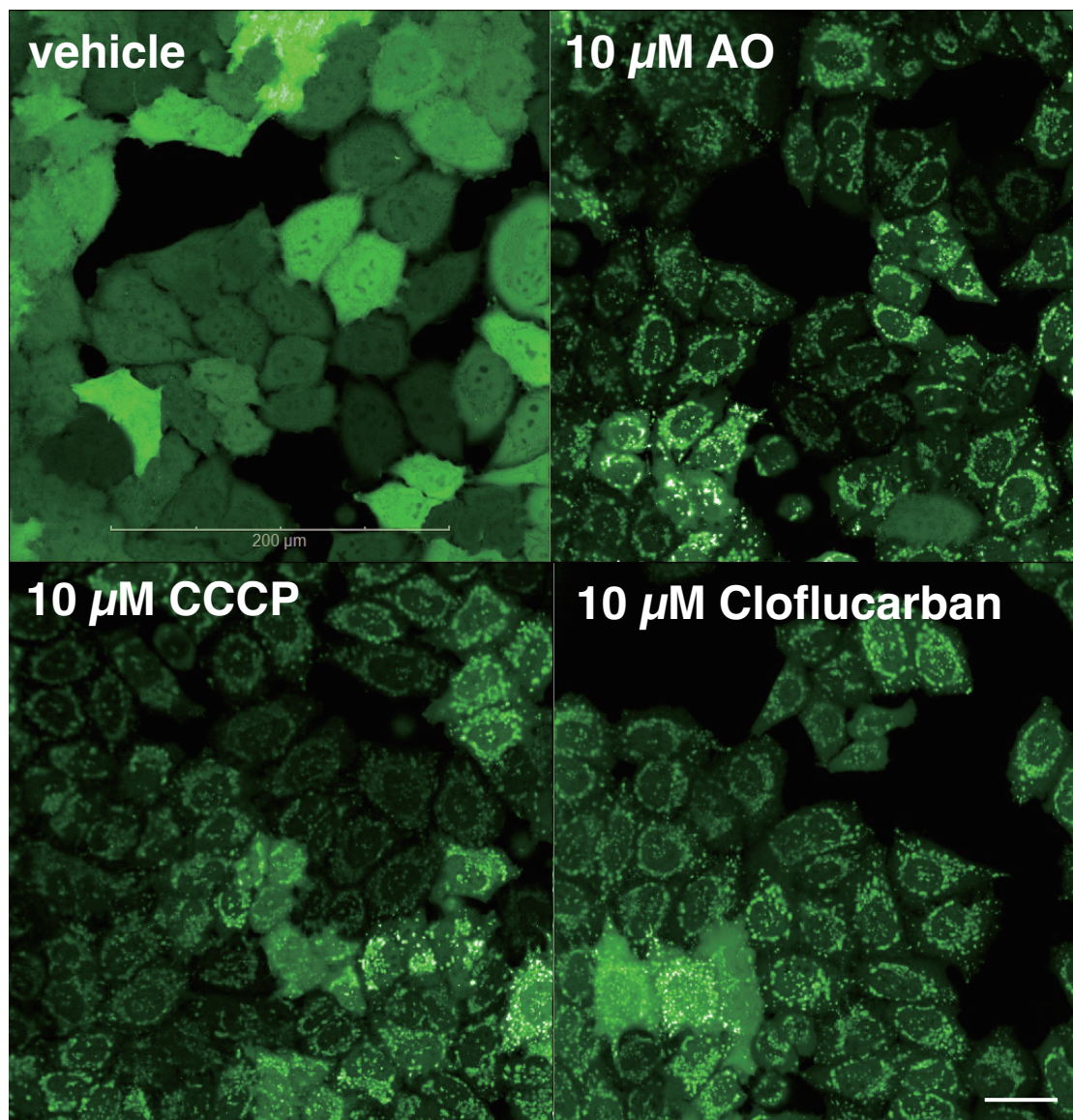**B**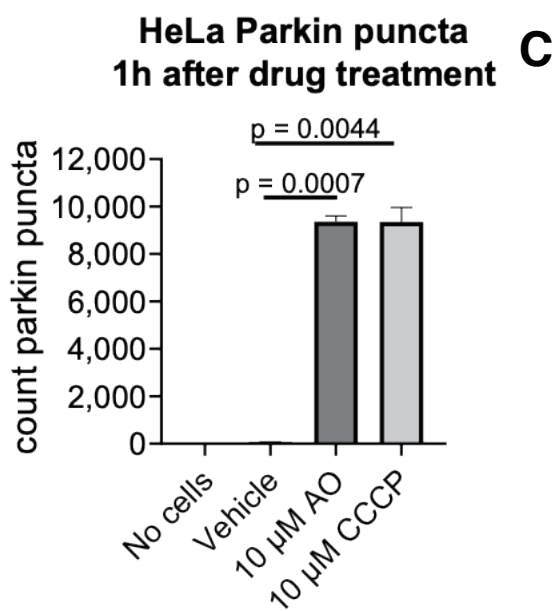**C**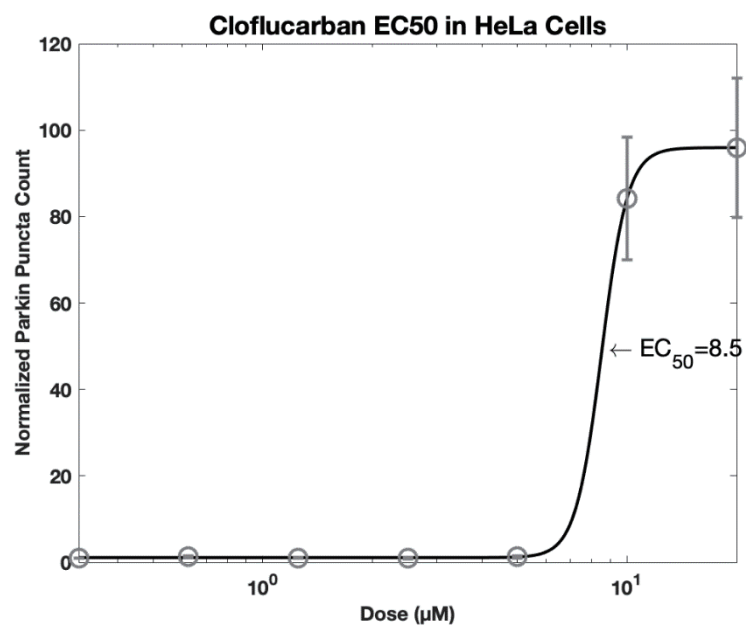

Supplement: Supplementary file 1 [file biomolecules-14-00248-s001.zip › Supplemental Figure S2.pdf]
